# Effects of Traditional Chinese Herbal Extracts on Tear Staining, Iron Status, Immune Function, and Antioxidant Capacity in Dogs

**DOI:** 10.3390/ani16111596

**Published:** 2026-05-24

**Authors:** Erdan Wang, Peng Wu, Jiu Xu, Xinrong Hong, Nian Zhao, Tao Sun, Tianfeng Zhang, Zhihao Xu, Caimei Yang

**Affiliations:** 1 Key Laboratory of Applied Technology on Green-Eco-Healthy Animal Husbandry of Zhejiang Province, College of Animal Science and Technology & College of Veterinary Medicine, Zhejiang Agricultural and Forestry University, Eastlake Campus, Hangzhou 311300, China; wangerdan@zafu.edu.cn (E.W.); w19939905069@163.com (P.W.); tsun@zafu.edu.cn (T.S.); zhangtf1203@163.com (T.Z.); xzhihao0220@163.com (Z.X.); 2 Shanghai Bridge PetCare Company, Shanghai 201506, China; jerry.xu@bpet.cn (J.X.); xinrong.hong@bpet.cn (X.H.); nian.zhao@bpet.cn (N.Z.)

**Keywords:** *Allobaculum*, *Cassia semen*, *Chrysanthemum morifolium*, dog, *Poria cocos*, tear staining

## Abstract

Tear staining is a common yet often underestimated condition in dogs that affects appearance and may reflect underlying disease. This study evaluated dietary supplementation with the traditional Chinese herbs *Chrysanthemum morifolium* (CM), *Cassia semen* (CS), and *Poria cocos* (PC) in dogs predisposed to tear staining and investigated potential mechanisms. Forty Poodle and Bichon dogs were fed diets supplemented with 0.5% CM, CS, or PC. Tear staining indices were measured biweekly, together with blood indicators of iron status, immune and inflammatory markers, and antioxidant capacity. All three supplements reduced tear staining (*p* < 0.05), as shown by lower scores and shorter stain length versus control. CM and PC improved iron status and porphyrin metabolism by increasing serum iron and ferritin and modulating δ-aminolevulinic acid and porphobilinogen deaminase. They also enhanced humoral immunity and antioxidant status by increasing serum immunoglobulins, reducing pro-inflammatory cytokines, and moderately improving antioxidant activities. CM and PC increased fecal *Allobaculum* abundance, which correlated with tear staining indices and porphyrin-related measures. In conclusion, CM, CS, and PC are promising dietary strategies for reducing tear staining in dogs, with CM and PC possibly acting via *Allobaculum*-associated modulation of iron and porphyrin metabolism, while improving iron status, immunity, and antioxidant defense.

## Simple Summary

Tear staining is a common yet underestimated condition that impairs appearance and may indicate underlying health issues in dogs. This study explored the effects of the traditional Chinese herbs *Chrysanthemum morifolium* (CM), *Cassia semen* (CS), and *Poria cocos* (PC) in dogs prone to tear staining. All three treatments reduced tear staining scores and stain length. Moreover, CS and PC improved iron and porphyrin status, immune function, and antioxidant capacity. Notably, CM and PC increased the abundance of genus *Allobaculum* in feces, which was associated with tear staining indices and porphyrin metabolites. These findings suggest that CM, CS, and PC may serve as promising dietary strategies for managing tear staining in dogs, while improving iron status, immunity, and antioxidant defense.

## 1. Introduction

Dog tear staining is a common yet often underestimated condition in canine care, characterized by reddish-brown periocular discoloration due to porphyrin-rich tears, and is particularly prevalent in light-coated breeds such as Bichon Frisés and Poodles [1]. These predisposed breeds frequently exhibit congenitally narrow or abnormally angled nasolacrimal ducts, which impair tear drainage even under physiological tear production, leading to tear overflow and periocular staining [2]. Beyond cosmetic concerns, chronic exposure to porphyrin-rich tears and a persistently moist periocular environment may promote skin irritation and secondary infection [2,3]. In addition to anatomical abnormalities, tear staining has been associated with ocular diseases, porphyrin homeostasis, and systemic inflammatory or oxidative conditions [4,5,6,7]. Therefore, tear staining may lead to predisposed local complications and signal underlying systematic health status beyond the suboptimal canine appearance.

Despite the significance in canine health, effective management of tear staining remains challenging. Besides the routine cosmetic control by periocular wiping or hair trimming [8], there is currently no universally effective or specific therapy for tear staining. Clinical approaches primarily focus on identifying and treating the underlying conditions, such as nasolacrimal duct obstruction, conjunctivitis, dacryocystitis, allergy, or other infectious diseases, through interventions such as nasolacrimal flushing and topical or systemic antibiotics when indicated [8,9,10]. Historically, oral administration of low-dose tylosin has been reported to reduce tear staining after several weeks of treatment [11,12]. However, growing regulatory restrictions and concerns about antimicrobial resistance have limited the use of antibiotics, and the intentional inclusion of antibiotics in commercial dog foods is generally considered inappropriate and uncommon in current practice [13]. Thus, tear staining management continues to rely largely on daily hygiene routines and dietary strategies aimed at improving overall health status. Collectively, these limitations underscore the need for safe, effective, and preferably natural supplements suitable for long-term management of canine tear staining.

Although studies conducted in dogs remain limited, traditional Chinese herbal ingredients have demonstrated beneficial effects on ocular disorders via coordinated modulation of inflammation, immunity, and redox balance in other models. *Chrysanthemum morifolium* (Ju Hua, CM) is a flavonoid-rich traditional Chinese herb and has been shown to alleviate retinal damage and dry eye-related lesions across animal models [14,15,16]. In rabbits, CM-derived flavonoids were found to relieve dry eye symptoms by inhibiting the proinflammatory cytokine expression and increasing TGF-β1 in lacrimal epithelial cells [15]. In rats, diosmetin, a key bioactive component from CM, protected against retinal injury and attenuated retinal cell apoptosis by limiting DNA damage and oxidative stress [14]. *Cassia Semen* (Jue Ming Zi, CS), literally translated to “seeds that can brighten the eyes” in Chinese, has long been used in traditional Chinese medicine for ocular health [17]. Research showed that aurantio-obtusin, a major bioactive compound in CS, alleviated dry eye disease in rats by inhibiting the NF-κB/NLRP3 inflammasome signaling pathway [18]. Another clinical study reported that drinking CS tea improved presbyopia, potentially through its antioxidant and pharmacological properties [19]. *Poria cocos* (Fu Ling, PC) is another widely used medicinal fungus in traditional Chinese medicine with major constituents including polysaccharides, triterpenoids, and other bioactive phytochemicals [20]. Polysaccharides and triterpenoids are the principal active components and have been reported to exert antioxidative, immunomodulatory, antitumor, hepatoprotective, and hypoglycemic effects [21]. Although direct evidence for ocular protection by PC is limited, these pharmacological properties may theoretically support ocular health and help ameliorate tear staining.

In addition to their reported ocular benefits, these herbal ingredients have been shown to exert immunomodulatory, antioxidant, and gut microbiota-modulating effects in various disease models, including alcohol-associated liver disease, Alzheimer’s disease, and diarrhea [22,23,24]. However, their efficacy in dogs, as well as their relevance to tear staining and its underlying mechanisms, remains largely unexplored. Therefore, the objective of the study was to explore the effectiveness of CM, CS, or PC in mitigating tear staining in dogs, and to investigate potential etiological mechanisms involving porphyrin and iron metabolism, immune system, inflammatory response, oxidative status, as well as intestinal microbial changes.

## 2. Materials and Methods

### 2.1. Animals, Diets, and Management

The study was performed in accordance with the protocols approved by the ethical committee of Zhejiang Agricultural and Forestry University for the use of animals (Protocol number: ZAFUAC2024009). The feeding trial was carried out from September to October 2025 in the Pet Research Base of Zhejiang Agricultural and Forestry University (Huzhou city, Zhejiang province, China).

At the beginning of the experiment, the age in months, body weight, and tear staining score (mean ± standard deviation) of the experimental dogs were found to be 21.45 ± 2.10 months, 3.79 ± 1.22 kg, and 2.66 ± 1.32, respectively. According to the principle of matching similar age, body weight and tear staining degree, twenty-eight Bichon Frisés and twelve Poodles were randomly assigned into one of four treatments: (1) control diet (CON), basal diet supplemented with 0.5% extrusion carriers (without any Chinese herbal extracts); (2) CM supplemented diet (CM), basal diet supplemented with 0.5% CM; (3) CS supplemented diet (CS), basal diet supplemented with 0.5% CS; (4) PC supplemented diet (PC), basal diet supplemented with 0.5% PC. Each group included 7 Bichon Frisés and 3 Poodles (n = 10 dogs per group) with comparable baseline month age, body weight, and tear staining severity (as presented in Table 1).

The experimental diets were formulated to meet the standards of Association of American Feed Control Officials (AAFCO) and manufactured by Shanghai Bridge PetCare Company (Shanghai, China). Detailed diet compositions are presented in Supplemental Table S1. The CM, CS and PC extracts (10:1) used in this study were purchased from Shaanxi Sinuote Bio-Tech Co. Ltd. (Baoji, China). The brief production process of the traditional Chinese herbal extracts was carried out in accordance with the Chinese Pharmacopoeia (2020 Edition). Fresh CM flowers and raw CS were cleaned to remove impurities and dead leaves; meanwhile, fresh PC was peeled manually to remove the outer skin and impurities, cut into small pieces (1–2 cm in diameter), and then dried in a constant-temperature drying oven until the moisture content was lower than 10%. After drying, the herbs were crushed and sieved through a 40-mesh sieve to obtain herb powders. The herb powder-to-purified water ratio was controlled to 1:10, reflux extraction was performed 2–3 times at 85–90 °C, 1–1.5 h each time. Filtration was conducted to remove herb residues and large particulate impurities, then fed into a spray drying tower to obtain herbal extract. The guaranteed active components of CM, CS and PC were total flavonoids (75%), total Anthraquinones (3.5%) and poria polysaccharides (35%), respectively.

All experimental dogs were vaccinated with Intervet Nobivac DHPPi for primary immunization. Dogs were housed individually in standard dog cages with dimensions of 90 cm (length) × 60 cm (width) × 75 cm (height), which was sufficient to allow the dogs to stand freely, turn around, and stretch their bodies comfortably. The environmental parameters of the feeding facility were strictly controlled: the ambient temperature was maintained at 18~26 °C, and the relative humidity was controlled at 40~70%. A program-controlled ventilation system was adopted for regular ventilation and air exchange every day to ensure that the ammonia concentration in the housing was lower than 15 ppm, and the air quality met the national standards for laboratory animal feeding environments. A standardized feeding management protocol was implemented daily: feces and cage dirt were cleaned regularly to keep the feeding environment clean and dry; drinking water was replaced daily to ensure that the dogs had free access to fresh and clean drinking water 24 h a day. Body condition scoring (BCS) was evaluated weekly using 9-point criteria as described in Supplemental Table S2, where scores of 1–3 indicate underweight, 4–5 indicate an ideal body condition, and 6–9 indicate overweight or obesity. All dogs maintained BCS within the ideal range during the whole period. Fecal scores were also assessed weekly following the criteria described in Supplemental Table S3.

### 2.2. Tear Staining Assessment

Tear staining was evaluated by following the scoring criteria described in Supplemental Table S4. The tear staining scoring of dogs adopted a 3-person independent blind evaluation method. Three trained evaluators scored all experimental dogs independently under the same light conditions and within the same time period, strictly following the unified tear staining scoring standard. The evaluators did not interfere with each other and did not exchange their scoring results. The final tear staining score for each individual dog was the average of the scores given by the three evaluators, which was then subjected to statistical analysis. Assessments were conducted at 7-day intervals starting on Day 7. At the beginning of each assessment period, tear stains were completely removed. Tear staining was scored at the end of each interval and then cleaned prior to the next assessment period. Changes in tear staining were calculated as the difference between baseline and end point scores for each interval. Other tear-related parameters, including tear secretion, pH, cytology and microbial culture, and tear staining length, were assessed in all dogs at 7-day intervals after the start of the study. Tear secretion was evaluated using the Schirmer tear test (STT1) to measure basal tear production [25,26]. Tear pH was determined using pH test strips (Shanghai SSS Reagent Co., Ltd., Shanghai, China). Tear staining length was measured using a ruler as the distance from the medial canthus to the distal edge of the stain. Measurements were obtained bilaterally and recorded for both eyes.

### 2.3. Blood Collection and Biochemical Analysis

Blood samples were collected from all dogs on Day 28 (the end of the intervention) via jugular venipuncture using a peripheral venous catheter [27]. Samples were centrifuged at 3000× *g* for 10 min at 4 °C to separate serum for subsequent analyses. Hematological, biochemical, and antioxidant parameters were then measured to evaluate the effects of dietary treatments on general physiological functions and immune status in dogs [28,29].

Hematological analysis was performed by following previous protocols to assess white blood cells (WBC), lymphocyte count, monocyte count, neutrophil count, lymphocyte percentage, neutrophil percentage, red blood cells (RBC), hemoglobin (HGB), hematocrit (HCT), mean corpuscular volume (MCV), mean corpuscular hemoglobin (MCH), mean corpuscular hemoglobin concentration (MCHC), red cell distribution width-coefficient of variation (RDW-CV), platelets (PLT), mean platelet volume (MPV), platelet distribution width (PDW), plateletcrit (PCT), and eosinophil percentage (EOS%) [30].

Serum biochemical parameters were determined using a fully automatic biochemical analyzer (Chengdu Smart Technology Co., Ltd., Chengdu, China), including albumin (ALB), total protein (TP), globulin (GLOB), albumin/globulin (A/B), total bilirubin (TB), γ-glutamyl transferase (GGT), aspartate aminotransferase (AST), alanine aminotransferase (ALT), alkaline phosphatase (ALP), total bile acids (TBA), amylase (AMY), lipase (LPS), lactate dehydrogenase (LDH), creatine kinase (CK), creatinine, uric acid, urea, urea/creatinine ratio (U/C), glucose, total cholesterol, triglycerides, total carbon dioxide, calcium, inorganic phosphorus, AST/ALT ratio, and calcium × phosphorus product.

Antioxidant indices, including malondialdehyde (MDA), superoxide dismutase (SOD), catalase (CAT), and glutathione peroxidase (GSH-Px), were measured using commercial assay kits following the manufacturers’ instructions (Aoqing, Nanjing, China). Serum immunoglobulins, including Immunoglobin A, M, and G (IgA, IgM, and IgG), and inflammatory cytokines, including tumor necrosis factor alpha (TNF-α), interleukin 1 beta (IL-1β), and interleukin 6 (IL-6), were evaluated by previously described protocols (Aoqing, Nanjing, China).

### 2.4. Iron Metabolism-Related Parameters

Iron metabolism-related parameters, including serum iron, total iron-binding capacity (TIBC), and serum ferritin, were measured using established analytical protocols and commercial assay kits [27,31]. Porphyrin metabolism-related indicators, including erythrocyte enzyme activity of porphobilinogen (PBG) deaminase and δ-aminolevulinic acid (ALA), were evaluated by using commercial assay kits (Aoqing, Nanjing, China).

### 2.5. Fecal Microbiome Analysis

Fecal samples were collected on Day 28 (the end of the intervention). The middle part of fresh feces (about 0.5 g) was collected using a sterile sampler and quickly aliquoted into labeled sterile enzyme-free cryovials and frozen at −80 °C until further analysis. Microbial genomic DNA was isolated from fecal samples using the E.Z.N.A.^®^ Soil DNA Kit (Omega Bio-tek, Norcross, GA, USA) following the manufacturer’s instructions. Sequencing libraries targeting the bacterial 16S rRNA gene were prepared and sequenced on the Illumina HiSeq platform, focusing on the V4 region. The V3–V4 hypervariable regions of the bacterial 16S rRNA gene were amplified using the primer pair 338F (5′-ACTCCTACGGGAGGCAGCAG-3′) and 806R (5′-GGACTACHVGGGTWTCTAAT-3′). PCR amplification was performed using a T100 Thermal Cycler (Bio-Rad, Hercules, CA, USA). Amplified products were separated on 2% agarose gels, excised, and purified using a commercial gel extraction kit (Yuhua, Shanghai, China). Purified amplicons were quantified with a Qubit 4.0 fluorometer (Thermo Fisher Scientific, Waltham, MA, USA). Raw sequencing reads were quality-filtered using fastp (v0.19.6) and merged with FLASH (v1.2.11). Operational taxonomic units (OTUs) were taxonomically assigned using a Naive Bayes classifier implemented in QIIME 2 against the SILVA 16S rRNA database, with a confidence threshold of 70%. Alpha diversity indices were calculated using Mothur (v1.30.2). Beta diversity was assessed based on unweighted UniFrac distances using QIIME (v1.9.1) and visualized through principal coordinate analysis (PCoA). Microbial community composition was summarized using the tax_summary script and analyzed with R software (v3.3.1). Differences in microbial taxa among experimental groups were evaluated using STAMP software (v2.1.3).

### 2.6. Statistical Analysis

All data were analyzed using SAS (version 9.4; SAS Institute Inc., Cary, NC, USA). The power analysis was conducted to estimate sample size before the feeding trial. According to the results of the power analysis, 40 dogs in a completely randomized design (n = 10) would be statistically sufficient with α (significance level) = 0.05 and power = 0.80 to show biologically relevant treatment differences for tear staining assessment and Serum biochemical parameters. The variables within one day or one sampling were analyzed using the PROC MIXED model, included the fixed effect of diet treatments and the random effect of the dogs as follows:

$$Y_{ij}\;=\;\mu \;+\;{\mathrm{Treat}}_{i}\;+\;{\mathrm{Dog}}_{j(i)}\;+\;E_{ij}$$

where *Y_ij_* = the dependent variable, *μ* = the overall mean, Treat*_i_* = the fixed effect of the *i*th diet treatments (*i* = 1 to 4), Dog_*j*(*i*)_ = the random effect of the *j*th dog fed the *i*th diet (*l* = 1 to 10), and *E_ijk_* = residual error, assumed to be normally, identically, and independently distributed (NIID). The best variance and covariance structure models were selected based on the values of the Akaike information criterion (AIC) and Bayesian information criterion (BIC). Results were presented as mean ± standard error. *p* < 0.05 indicates significant difference. Differences were considered significant at *p* ≤ 0.05, highly significant at *p* < 0.01, and tendency at 0.05 < *p* ≤ 0.10.

## 3. Results

### 3.1. Effects of Dietary Treatments on Tear Staining and General Health

**Figure 1:**
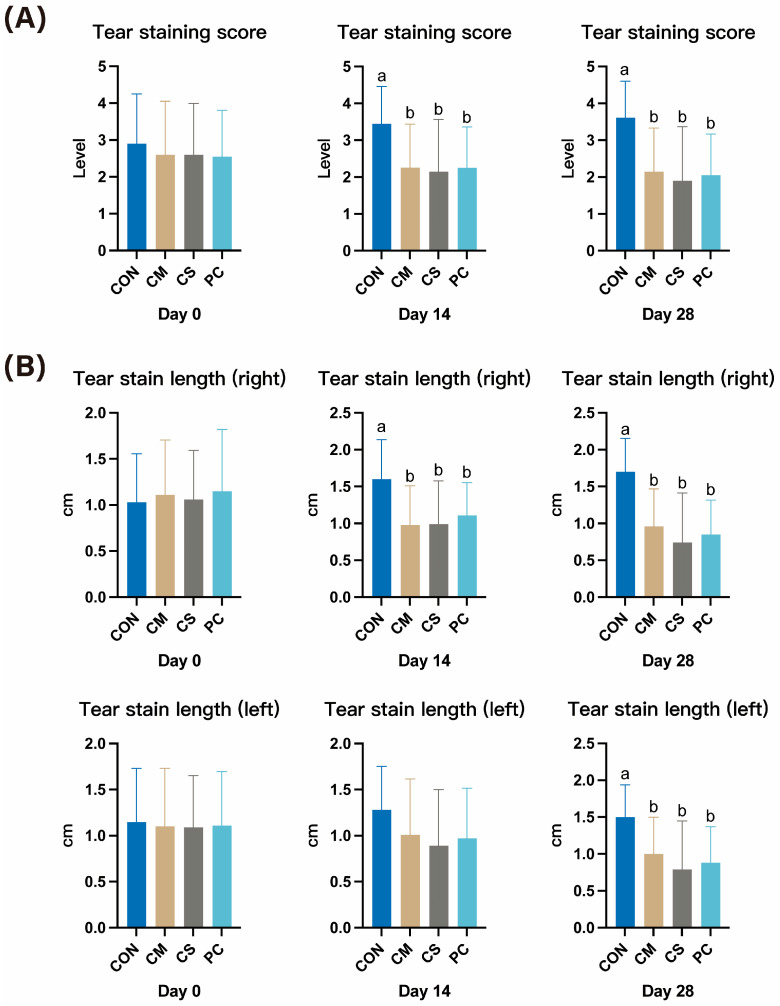
Effects of dietary supplementation with Chinese herbal extracts on tear staining score (**A**) and tear stain length of right and left eye (**B**) at days 0, 14, and 28. Values with different superscript letters represent significant contrast differences (*p* < 0.05).

**Figure 2:**
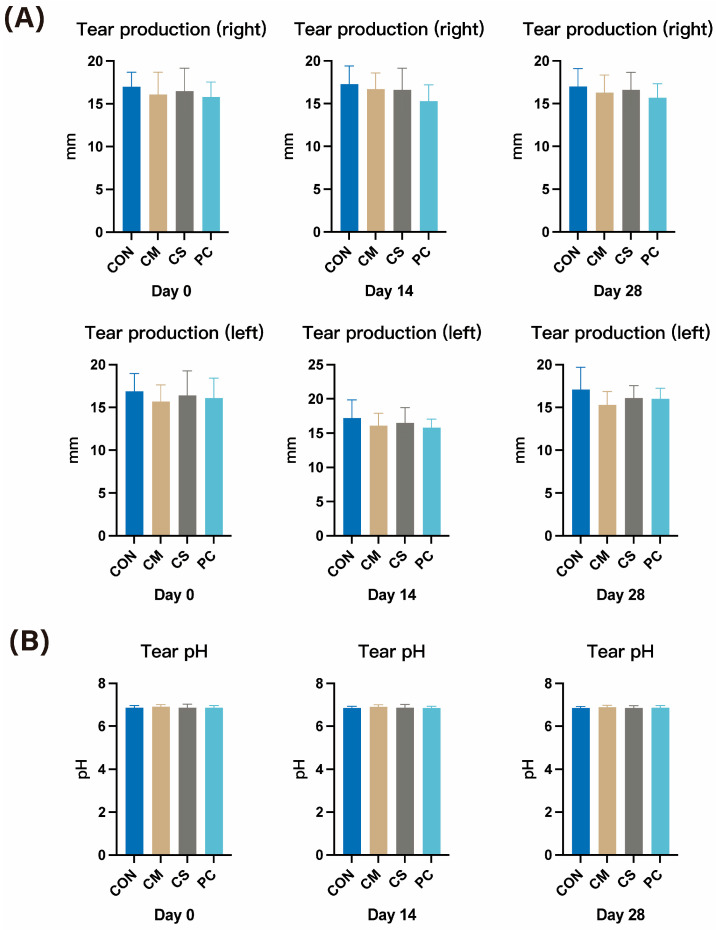
Effects of dietary supplementation with Chinese herbal extracts on tear production of right and left eye (**A**) and tear pH (**B**) at days 0, 14, and 28.

Tear staining score and tear stain length at days 0, 14, and 28 are presented in Figure 1. All dietary treatments including CM, CS, and PC decreased (*p* < 0.05) tear staining score by 38% to 49% compared with the control at both days 14 and 28. Tear stain length decreased consistently with that in tear staining score. Although there was a discrepancy of tear stain length between right and left eyes at day 14, both eyes showed improvement (*p* < 0.05) at day 28, with tear stain length reduced (33–57%) by CM, CS, and PC over CON. In contrast, tear production from both eyes and tear pH did not differ among treatments presented in Figure 2. In addition, general health related parameters, including fecal score, body condition score, and body weight did not differ among treatments over the experimental period (Supplemental Tables S5–S7).

### 3.2. Effects of Dietary Treatments on Porphyrin Metabolism and Iron Status

**Figure 3:**
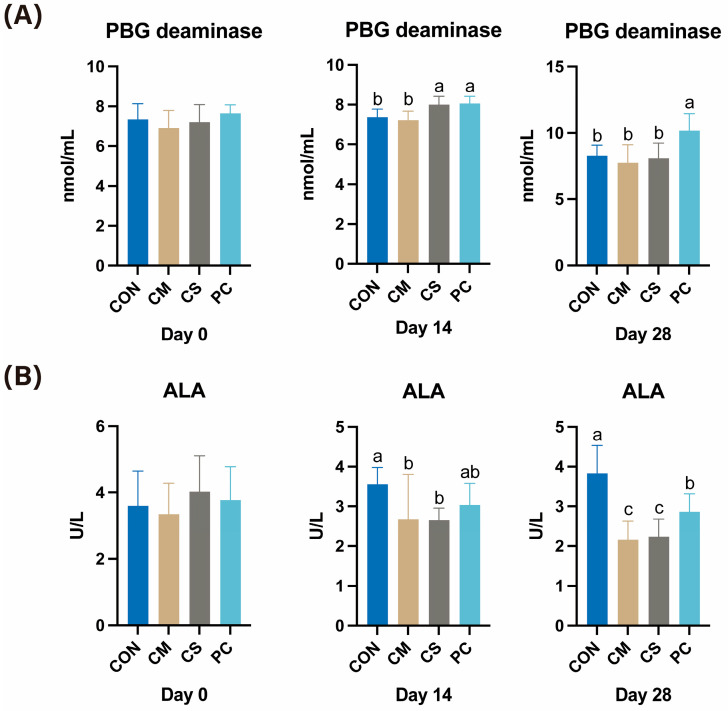
Effects of dietary supplementation with Chinese herbal extracts on PBG deaminase (**A**) and ALA (**B**) at days 0, 14, and 28. PBG deaminase = porphobilinogen deaminase activity; ALA = δ-aminolevulinic acid activity. Values with different superscript letters represent significant differences (*p* < 0.05).

Porphyrin metabolism-related indicators are shown in Figure 3. At day 14, CS and PC increased PBG deaminase activity by 8.4–9.4% (*p* < 0.05) compared with the control, whereas only PC maintained a higher PBG deaminase activity (*p* < 0.05, 23%) at day 28. In contrast, ALA activity was 25 to 26% lower (*p* < 0.05) in the CM and CS groups than in the control at day 14, and by day 28 all three treatments (CM, CS, and PC) showed reduced ALA activity (*p* < 0.05, 25–44%) relative to the control, with CM and CS exhibiting even lower ALA activity than PC.

**Figure 4:**
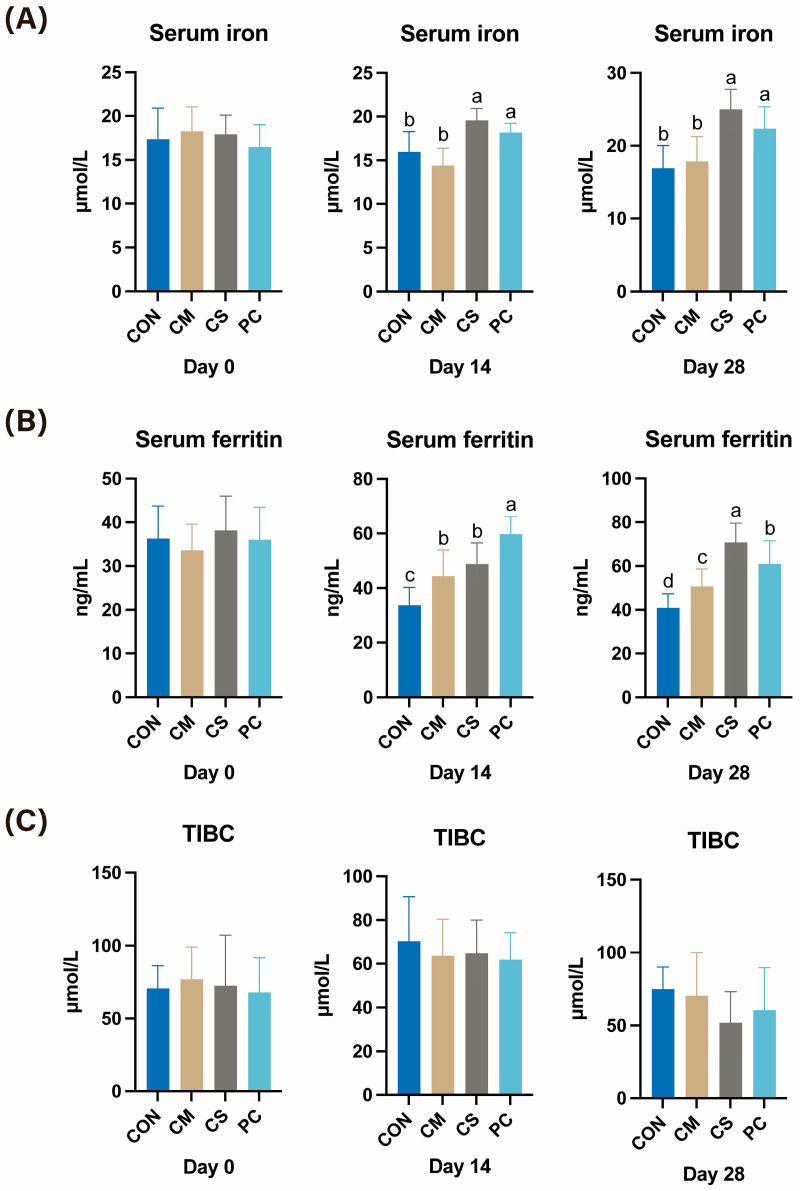
Effects of dietary supplementation with Chinese herbal extracts on Serum iron (**A**), Serum ferritin (**B**) and TIBC (**C**) at days 0, 14, and 28. TIBC = total iron-binding capacity. Values with different superscript letters represent significant differences (*p* < 0.05).

Serum iron status including serum iron and ferritin displayed a similar increasing trend shown in Figure 4. At day 14, CS and PC treatments increased (*p* < 0.05) serum iron concentrations by 14–23% compared with CON, and this difference (*p* < 0.05) was further expanded to 32–48% at day 28. Meanwhile, all three treatments (CM, CS, and PC) resulted in higher serum ferritin (*p* < 0.05, 24–77%) than the control at days 14 and 28, with PC showing the highest at day 14 and CS at day 28. Despite these increases in serum iron and ferritin, TIBC remained unchanged throughout the experimental period among treatments.

### 3.3. Immune Function and Oxidative Status

**Figure 5:**
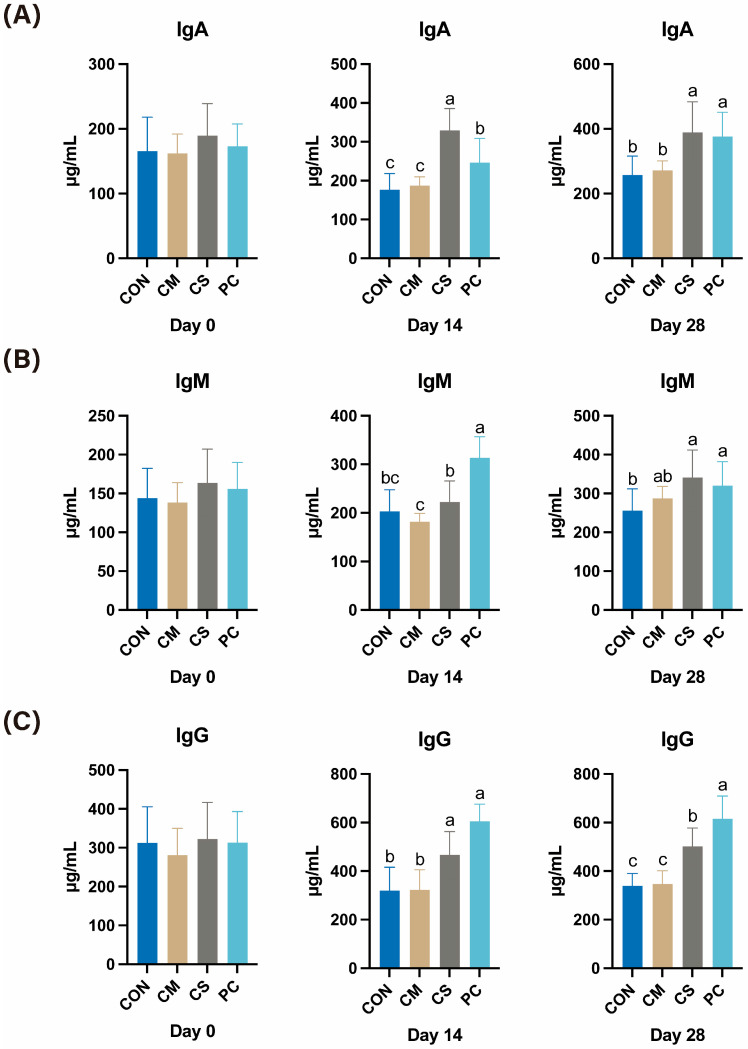
Effects of dietary supplementation with Chinese herbal extracts on IgA (**A**), IgM (**B**) and IgG (**C**) at days 0, 14, and 28. Values with different superscript letters represent significant differences (*p* < 0.05).

**Figure 6:**
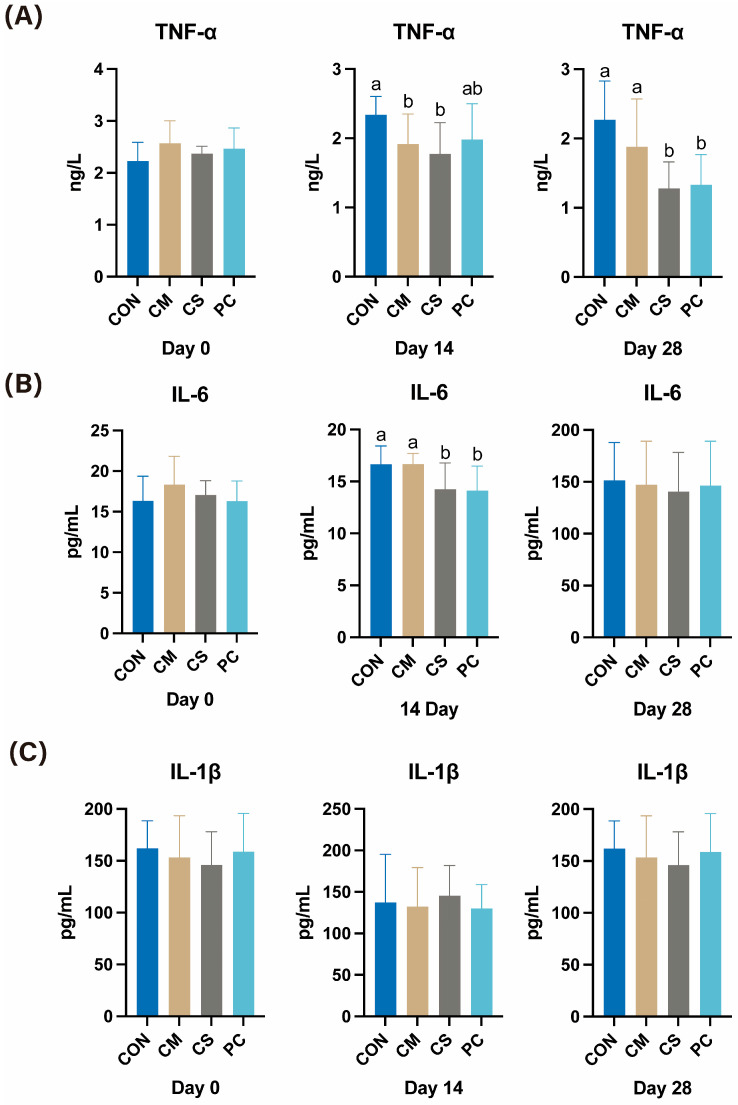
Effects of dietary supplementation with Chinese herbal extracts on serum TNF-α (**A**), IL-6 (**B**) and IL-1β (**C**) at days 0, 14, and 28. Values with different superscript letters represent significant differences (*p* < 0.05).

The concentrations of serum immunoglobulins and cytokines are shown in Figure 5 and Figure 6. All immunoglobulins were increased mainly by the CS and PC treatments but not by CM. At days 14 and 28, serum IgA and IgG concentrations were higher (*p* < 0.05, 25–87%) in CS and PC groups than in CON and CM. In addition, only PC increased (*p* < 0.05, 54%) IgM at day 14 compared with CON, whereas at day 28 both CS and PC treatments showed a higher (*p* < 0.05) IgM concentration by 33% and 25% than the control, respectively. Cytokine concentrations largely paralleled the immunoglobulin responses. At day 14, CM and CS reduced (*p* < 0.05, 18–24%) serum TNF-α compared with the control, and by day 28 TNF-α was further decreased (*p* < 0.05) in the CS and PC groups by 41–44% relative to CON, while no difference was found between CM and CON. Meanwhile, IL-6 concentrations in the CS and PC were 14–15% lower (*p* < 0.05) than those in the CON at day 14, with no difference at day 28.

### 3.4. Antioxidant Status

**Figure 7:**
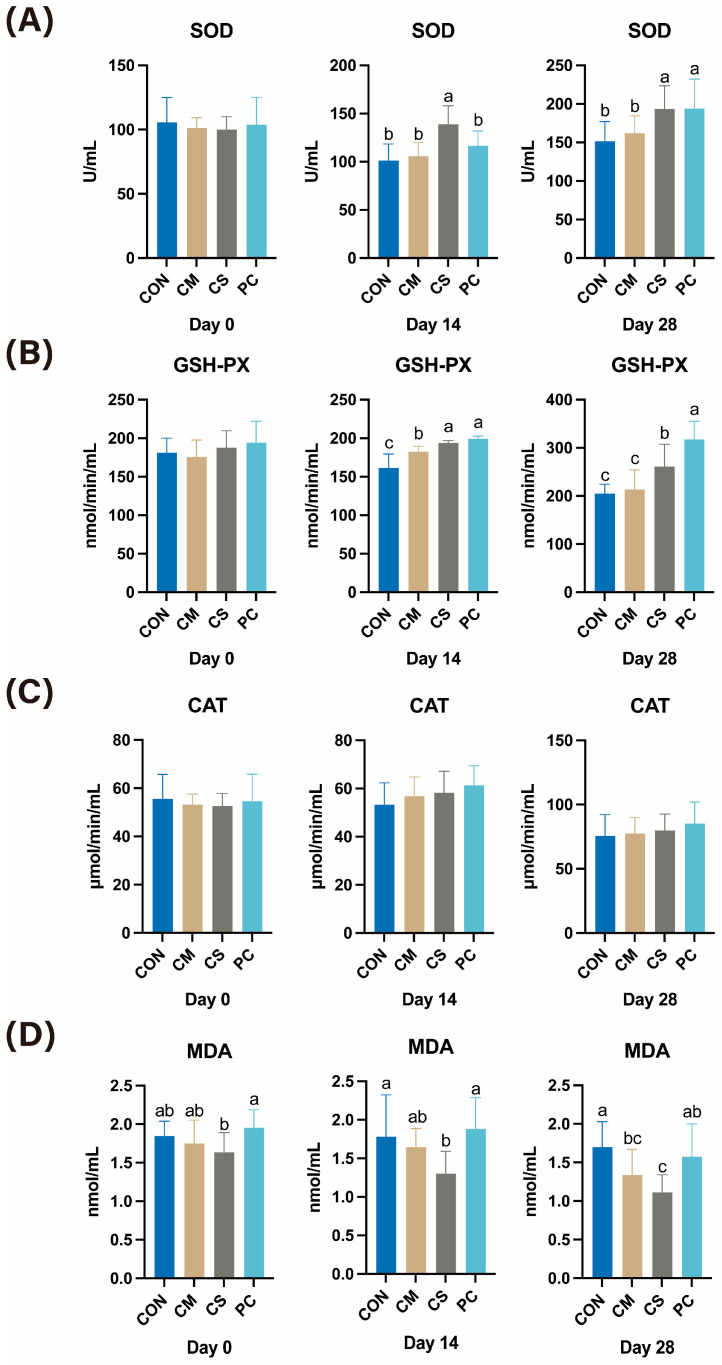
Effects of dietary supplementation with Chinese herbal extracts on serum SOD (**A**), GSH-Px (**B**), CAT (**C**) and MDA (**D**) at days 0, 14, and 28. Values with different superscript letters represent significant differences (*p* < 0.05).

Antioxidant status was assessed by serum antioxidant enzyme activities and MDA concentrations (Figure 7). Overall, the SOD, GPX, and CAT activities were increased significantly or with a trend in dietary supplementations with CM, CS and PC when compared with CON throughout the trial. For SOD, only CS showed an increase (*p* < 0.05, 37%) at day 14, whereas both CS and PC were higher (*p* < 0.05) than CON by 28% at day 28. No significant changes were observed in CM. GPH-PX activity was elevated (*p* < 0.05, 13–23%) in all dietary supplement groups at day 14, while only CS and PC groups remained higher (*p* < 0.05) than CON by 28% and 55% at day 28, respectively. Although CAT activity did not differ significantly among groups, all supplemented groups exhibited higher values by 2.6–15% than CON. In parallel to enhanced antioxidant activities, the CS group showed a lower (*p* < 0.05, 27%) MDA concentration compared with the control at day 14 and 28, while no differences were observed for CM and PC.

### 3.5. General Blood Analysis Parameters

Hematological analysis and serum biochemical parameters are listed in the Supplemental Tables S8 and S9, respectively. Hematological analysis included white blood cells (WBC), lymphocyte count, monocyte count, neutrophil count, lymphocyte percentage, neutrophil percentage, red blood cells (RBC), hemoglobin (HGB), hematocrit (HCT), mean corpuscular volume (MCV), mean corpuscular hemoglobin (MCH), mean corpuscular hemoglobin concentration (MCHC), red cell distribution width-coefficient of variation (RDW-CV), platelets (PLT), mean platelet volume (MPV), platelet distribution width (PDW), plateletcrit (PCT), and eosinophil percentage (EOS%). In addition, serum biochemical parameters include albumin (ALB), total protein (TP), globulin (GLOB), albumin/globulin (A/B), total bilirubin (TB), γ-glutamyl transferase (GGT), aspartate aminotransferase (AST), alanine aminotransferase (ALT), alkaline phosphatase (ALP), total bile acids (TBA), amylase (AMY), lipase (LPS), lactate dehydrogenase (LDH), creatine kinase (CK), creatinine, uric acid, urea, urea/creatinine ratio (U/C), glucose, total cholesterol, triglycerides, total carbon dioxide, calcium, inorganic phosphorus, AST/ALT ratio, and calcium × phosphorus product. No differences were found between treatments from all parameters.

### 3.6. Effects of the Supplements on the Microbial Diversity of Dogs

**Figure 8:**
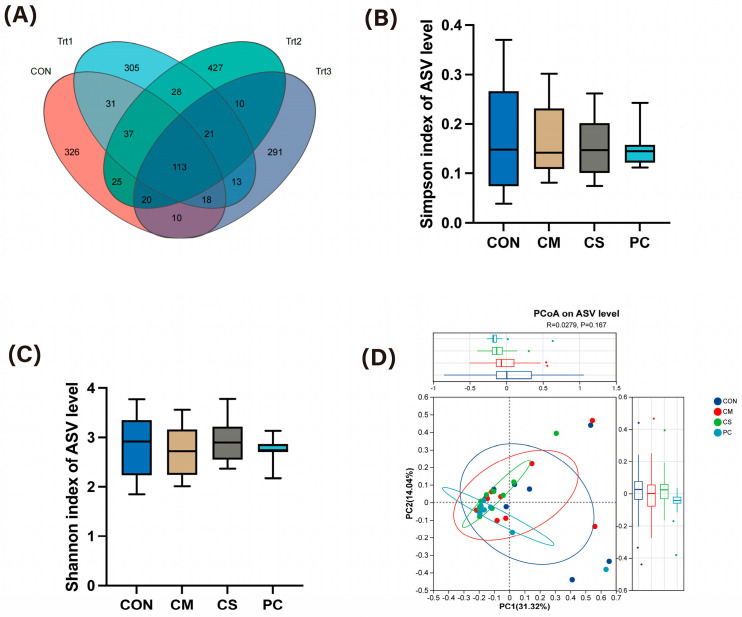
Effects of dietary supplementation with Chinese herbal extracts on fecal microbiota ASV distribution (**A**), Shannon indexes (**B**), Simpson indexes (**C**) and PCoA (**D**) at day 28.

16S rRNA sequencing was performed to characterize the fecal microbiota of dogs on day 28 at the ASV levels shown in Figure 8A. Venn diagram analysis showed that 113 common ASVs were shared among all four groups, while the CON, CM, CS and PC groups had 326, 305, 427, 291 unique ASVs, respectively, supporting the treatment related shifts in the individual microbial taxa. Alpha diversity was then assessed by Shannon and Simpson indexes. As shown in Figure 8B,C, there was no difference in either index among four groups, indicating comparable within-sample microbial diversity across treatments. Beta diversity was evaluated using principal coordinates analysis (PCoA) plot (Figure 8D). Samples among all groups largely overlapped in ordination space with only a few outliers, suggesting no significant differences in overall community structure and composition. Therefore, the general microbial diversity of dogs remained the same despite dietary treatments.

### 3.7. Effects of the Supplements on the Microbial Composition of Dogs and Correlation Analysis

**Figure 9:**
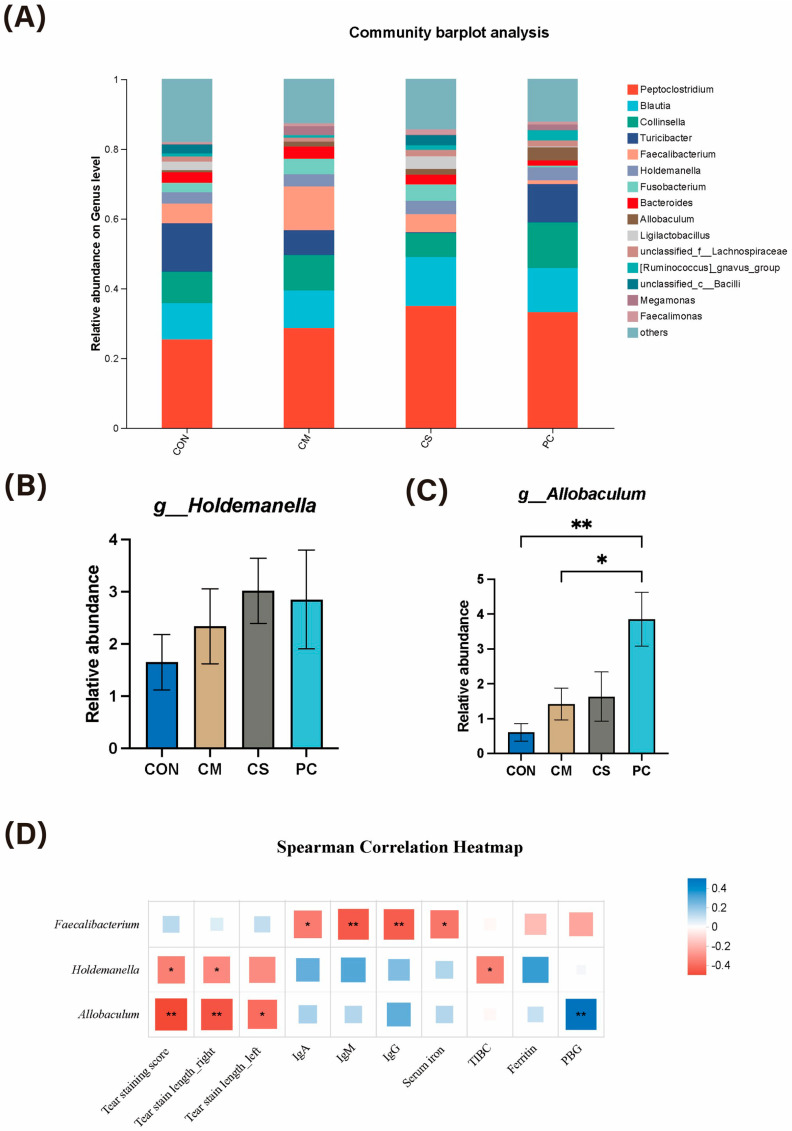
Effects of dietary supplementation with Chinese herbal extracts on fecal microbial composition at genus level (**A**), relative abundance of *g_Holdemanella* (**B**), relative abundance of *g_Allobaculum* (**C**), and a correlation analysis between differential genera and differential tear staining parameters (**D**). * represents a significant differences (*p* < 0.05), ** represents a highly significant differences (*p* < 0.01).

The fecal microbial composition indicated that Peptoclostridium, Blautia, Collinsella, Turicibacter, Faecalibacterium, Holdemanella, Fusobacterium, Bacteroides, and Allobaculum were the dominant bacteria genera in the dogs (Figure 9A). In addition, Figure 9B,C illustrates differences in relative abundance at the genus level. The relative abundance of Allobaculum was higher (*p* < 0.05) in the CM and PC groups over the control, whereas CS also showed an increased trend that did not reach significance. Correlations between fecal genera and serum indicators including tear staining parameters, immune response, iron status, and antioxidant markers were further evaluated by Spearman correlation analysis (Figure 9D). Holdemanella and Allobaculum were negatively correlated (*p* < 0.05) with tear staining score and tear stain length, and both genera were also associated with iron or porphyrin indicators. Specifically, Holdemanella was negatively correlated (*p* < 0.05) with TIBC, whereas Allobaculum was positively correlated (*p* < 0.05) with PBG deaminase. Moreover, Faecalibacterium was negatively associated (*p* < 0.05) with serum iron immunoglobins including IgA, IgM, and IgG. Overall, dietary supplements modulated the abundance of several genera, which were also correlated with tear staining parameters, iron or porphyrin status, or immunoglobins.

## 4. Discussion

Tear staining is a seemingly minor but commonly observed issue in dogs. In the present study, genetically predisposed breeds (Bichon Frisés and Poodles) were recruited due to their relatively narrow or angled nasolacrimal ducts [2]. Those breeds are more susceptible to porphyrin deposition and a persistently damp periocular environment, which may lead to secondary microbial infections [2,3]. Besides, tear staining may reflect underlying infectious, inflammatory, or oxidative stress-related conditions [4,5,6,7]. To date, there is no consistently effective and non-antibiotic treatment targeting tear staining, and routine management including periocular cleaning and hair trimming remains necessary [8]. In this study, the ingredients derived from traditional Chinese medicine have been explored. Our results found that supplementing CM, CS, or PC significantly alleviated tear staining, shown by the reductions in both subjective (tear staining scores) and objective (tear stain length) assessments after 28 days of feeding. Overall, our results suggested that all the dietary supplements could effectively improve tear staining in dogs.

The pigment in tear staining primarily consists of oxidized iron from porphyrin-containing compounds [32,33]. Porphyrins are tetrapyrrolic molecules that play key roles in heme and hemoglobin metabolism, serving as intermediates in heme synthesis and as byproducts of hemoglobin degradation, thereby linking erythrocyte turnover to systemic porphyrin and iron load [34]. Besides, porphyrins are mainly excreted via bile into the intestine; however, in dogs, a small amount can also be secreted through tears, saliva, and urine [35]. Hence, altered porphyrin metabolism or systemic iron status can influence the serum porphyrin concentrations, leading to increased excretion via tears and thereby contributing to tear staining. In the present study, all three dietary treatments including CM, CS, and PC significantly decreased the concentration of δ-aminolevulinic acid (ALA), the first intermediate in the heme biosynthetic pathway [36]. ALA is primarily synthesized in the liver and bone marrow, and its concentration reflects the initial rate of heme formation [37]. A reduction in ALA levels suggested a decreased flux through the heme biosynthetic pathway, leading to lower productions of downstream intermediates such as porphobilinogen-derived porphyrinogens and porphyrins. In the heme synthesis, porphobilinogen (PBG) deaminase catalyzes the conversion of PBG to hydroxymethylbilane and represents a pivotal mid-pathway enzyme in heme biosynthesis [36]. In the present study, the PC treatment increased PBG deaminase activity compared with the control at day 28. Therefore, enhanced PBG deaminase activity suggests that once PBG is formed, it is more efficiently converted to downstream products, reducing the risk of PBG and related intermediates accumulating. Collectively, these findings indicated the dietary supplementations did not exacerbate the accumulation of heme precursors (ALA, PBG) but instead reduced upstream pathway pressure while enhancing mid-pathway enzyme capacity, promoting a more balanced heme-porphyrin synthesis.

Besides porphyrin-related outcomes, iron status data further supported the improvement in tear staining. Since heme consists of a porphyrin ring coordinated with ferrous iron, iron status is closely related to porphyrin production [34]. Iron availability regulates porphyrin synthesis by modulating the rate-limiting enzyme ALA synthase [36,38]. Iron deficiency disrupts iron insertion into protoporphyrin IX and results in porphyrin accumulation and excretion, whereas adequate iron promotes heme synthesis and limits excess porphyrins [39]. Our results indicated that, at day 28, dogs in the CS and PC groups exhibited higher serum iron and ferritin concentrations, whereas total iron-binding capacity (TIBC) remained unchanged. The increase in serum iron and ferritin concentrations without inflammation indicated a larger circulating pool of bioavailable iron for hemoglobin synthesis and other iron-dependent processes [38,40]. Consistently, the unchanged TIBC suggested stable transferrin concentration and maximal iron-binding capacity [41]. Together, our findings suggested increased iron availability and storage, supporting more efficient porphyrin metabolism.

Changes in porphyrin and iron metabolism were accompanied by alterations in immune and oxidative status. Serum immunoglobulins including IgA, IgG, and IgM reflect humoral immune capacity by mediating antibody-dependent defense against extracellular pathogens and toxins in blood, lymph, and mucosal secretions [42]. Cytokines such as IL-1, IL-6, and TNF-α mainly produced by monocytes and macrophages, play roles in regulating inflammatory response by promoting immune cell recruitment and activation [43,44]. At day 28, dogs in the CS and PC groups exhibited increased serum IgA, IgM, and IgG concentrations, indicating enhanced humoral immune responses. In parallel, CS and PC decreased TNF-α, suggesting attenuation of low-grade inflammation and immune stress. Taken together, the combination of lower TNF-α with moderately elevated immunoglobulins is more consistent with a better regulated and less inflamed immune status rather than chronic inflammatory activation. Similarly, CS and PC tended to improve several oxidative stress-related indices, including higher activities of antioxidant enzymes such as SOD and GPx and/or lower levels of lipid peroxidation, although these effects were limited to a subset of parameters within each treatment [45,46]. Overall, these data indicated that CS and PC, but not CM, exerted modest but favorable effects on immune and antioxidant functions, which may contribute to the observed reduction in tear staining.

Previous studies have shown that Chinese herbal medicines could beneficially modulate the gut microbiota, but the associations between tear staining and gut microbes have not been reported extensively [22,24,47,48,49]. In our study, none of the three dietary supplementations altered microbial alpha or beta diversity, indicating that overall community richness and structure remained stable. However, PC and CM increased the relative abundance of the genus *Allobaculum*, and correlation analyses revealed that the abundance of *Allobaculum* and *Holdemanella* was significantly associated with tear staining parameters, as well as TIBC and PBG deaminase activity, respectively. Given the iron status is physiologically linked to tear staining, our results suggested a potential connection between these genera and tear staining via iron/porphyrin metabolism. Interestingly, both *Allobaculum* and *Holdemanella* are commensal Firmicutes with reported butyrate-producing capacity, fermenting dietary carbohydrates to yield short chain fatty acids [50,51]. Butyrate has been shown to lower intestinal pH, thereby enhancing iron solubility by increasing the reduction of ferric to ferrous ion and increasing intestinal absorptive capacity [52,53,54], consistent with the elevated serum iron and ferritin described above. Improved iron availability could reduce the demand for heme synthesis and consequently lower the requirement for de novo porphyrin production [36,55]. Meanwhile, intestinal butyrate has been reported to promote regulatory T cells differentiation, suppress inflammation, enhance IgA production and mucosal immune functions [56,57], which also aligned with higher immunoglobulins and lower pro-inflammatory cytokines in the herbal supplementation groups. Collectively, we hypothesize that the expansion of *Allobaculum* and related butyrate-producing taxa might have modulated porphyrin metabolism via gut-liver axis, thereby altering the heme synthesis demand and PBG deaminase activity. Such changes could reduce excess porphyrin burden and its elimination via tears, ultimately contributing to the improvement in tear staining observed in dogs. However, this proposed mechanism remains speculative without direct mechanistic evidence, as the study only identified correlations between microbial alterations and tear staining parameters without measurements of intestinal butyrate concentrations or validation through fecal microbiota transplantation experiments. Nevertheless, future studies including fecal microbiota transplantation, metabolic profiling, and short-chain fatty acid (SCFA) quantification are warranted to provide evidence for this interpretation.

## 5. Conclusions

Our study demonstrated that dietary supplementation with 0.5% *Chrysanthemum morifolium* (CM), cassia semen (CS), or *Poria cocos* (PC) alleviated dog tear staining after 28 days of feeding, highlighting their potential as management strategies. These benefits were associated with improvement of porphyrin/iron metabolism, immunity, and antioxidant capacity, and might partially involve alteration in gut microbiota, including increased abundance of *Allobaculum* and related butyrate-producing taxa. However, the study has limitations as the proposed gut microbiota-related mechanism remained preliminary and lacked causal evidence. The absence of consistent responses across all herbal groups suggested that CM, CS, and PC might act through distinct mechanisms or shared pathways with differing magnitudes of effects. Therefore, future trials with detailed chemical characterization of the herbal extracts and targeted investigation of gut microbiota and butyrate interactions are warranted to better elucidate their roles in minimizing tear staining.

## Figures and Tables

**Table 1:** Age, body weight, and tear staining score of the experimental dogs.

Group	Number of Dogs (n)	Age (Month)	Body Weight (kg)	Tear Staining Score
CON	10	21.50 ± 2.37	3.66 ± 0.99	2.90 ± 1.35
CM	10	21.60 ± 2.12	3.92 ± 1.47	2.60 ± 1.45
CS	10	21.00 ± 2.36	3.86 ± 1.67	2.60 ± 1.39
PC	10	21.70 ± 1.77	3.73 ± 0.73	2.55 ± 1.26
Total	40	21.45 ± 2.10	3.79 ± 1.22	2.66 ± 1.32

## Data Availability

The 16S rRNA gene sequencing data in the study were uploaded to the China National Center for Bioinformation (CNCB, http://www.cncn.ac.cn) with the accession number PRJNA1419741.

## Supplementary Materials

The following supporting information can be downloaded at: https://www.mdpi.com/article/10.3390/ani16111596/s1, Table S1. Nutrient compositions of the experimental diets; Table S2. Body condition score (BCS) grading criteria; Table S3. Fecal Scoring Criteria; Table S4. Tear staining grading criteria; Table S5. Effects of dietary supplements on fecal score assessment in experimental dogs; Table S6. Effects of dietary supplements on BCS in experimental dogs; Table S7. Effects of dietary supplements on body weight in experimental dogs; Table S8. Effects of dietary supplements on hematological parameters in experimental dogs; Table S9. Effects of dietary supplements on serum biochemical parameters in experimental dogs.

## Abbreviations

The following abbreviations are used in this manuscript:

CM	*Chrysanthemum morifolium*
CS	*Cassia semen*
PC	*Poria cocos*
TGF-β1	Transforming growth factor-beta 1
NF-κB	Nuclear factor kappa-B
NLRP3	NOD-like receptor family pyrin domain-containing 3
CON	control
BCS	body condition score
STT1	Schirmer tear test
WBC	white blood cells
RBC	red blood cells
HGB	hemoglobin
HCT	hematocrit
MCV	mean corpuscular volume
MCH	mean corpuscular hemoglobin
MCHC	mean corpuscular hemoglobin concentration
RDW-CV	red cell distribution width-coefficient of variation
PLT	platelets
MPV	mean platelet volume
PDW	platelet distribution width
PCT	plateletcrit
EOS%	eosinophil percentage
ALB	albumin
TP	total protein
GLOB	globulin
A/B	albumin/globulin
TB	total bilirubin
GGT	γ-glutamyl transferase
AST	aspartate aminotransferase
ALT	alanine aminotransferase
ALP	alkaline phosphatase
TBA	total bile acids
AMY	amylase
LPS	lipase
LDH	lactate dehydrogenase
CK	creatine kinase
U/C	urea/creatinine ratio
MDA	malondialdehyde
SOD	superoxide dismutase
CAT	catalase
GSH-Px	glutathione peroxidase
IgA, IgM, IgG	immunoglobin A, M, and G
TNF-α	tumor necrosis factor alpha
IL-1β	interleukin 1 beta
IL-6	interleukin 6
TIBC	total iron-binding capacity
PBG	porphobilinogen
ALA	δ-aminolevulinic acid
