# Supplementary material for: Effects of Traditional Chinese Herbal Extracts on Tear Staining, Iron Status, Immune Function, and Antioxidant Capacity in Dogs
Source: Animals (Basel). 2026 May 24;16(11):1596. doi: 10.3390/ani16111596 (PMC13255681; doi:10.3390/ani16111596)
Supplement: Supplementary file 1 [file animals-16-01596-s001.zip › animals-4251425-supplementary.pdf]

animals

MDPI

Supplemental Table S1. Nutritional composition of the experimental diets[1]

Ingredients (g/kg)	Control	CM	CS	PC
Deboned duck meat	400	400	400	400
Duck meat powder	150	150	150	150
Duck fat	40	40	40	40
Fish oil	20	20	20	20
Schizochytrium oil	1	1	1	1
Freeze-dried chicken meat	20	20	20	20
Freeze-dried egg yolk	20	20	20	20
Freeze-dried duck meat	20	20	20	20
Cucumber powder	10	10	10	10
Bitter melon powder	10	10	10	10
Loofah powder	10	10	10	10
Winter melon powder	10	10	10	10
Pumpkin powder	10	10	10	10
Zucchini powder	10	10	10	10
Goji berry	1	1	1	1
Extrusion carriers[2]	200	195	195	195
Premix[3]	68	68	68	68
Chrysanthemum morifolium		5		
Cassia Semen			5	
Poria cocos				5
Total	1000	1000	1000	1000

[1]Experimental diets were prepared by supplementing the control diet with *Chrysanthemum morifolium* (CM), Cassia semen (CS), or *Poria cocos* (PC), respectively.

[2]Extrusion carriers: tapioca starch, dried sweet potato, and peas.

[3]Premix: glucosamine hydrochloride, 1g/kg; sodium hyaluronate, 500 mg/kg; *Pediococcus acidilactici* (inactivated, postbiotic), 500 mg/kg; fructooligosaccharides (prebiotic), 500 mg/kg; vitamin A; vitamin D_3; dl-α-tocopherol acetate; thiamine nitrate (vitamin B_1); riboflavin (vitamin B_2); pyridoxine hydrochloride (vitamin B_6); cyanocobalamin (vitamin B_{12}); D-biotin; folic acid; niacinamide; D-calcium pantothenate; choline chloride; natural lutein (from marigold), 500 mg/kg; β-carotene, 100 mg/kg; zinc sulfate; copper sulfate; ferrous sulfate; manganese sulfate; sodium selenite; calcium iodate; potassium chloride; L-lysine hydrochloride; taurine, 1 g/kg; chondroitin sulfate; natural steroidal saponins (from Yucca schidigera); rosemary extract (natural antioxidant); tert-butylhydroquinone (TBHQ); and dicalcium phosphate.

Supplemental Table S2. Body condition score (BCS) grading criteria.

Grade	Criteria
BCS1	Extremely thin. From a distance, ribs, lumbar vertebrae, pelvic bones, and entire skeletal structure are clearly visible. Muscle mass is significantly insufficient, with a noticeably thin physique.
BCS2	Very thin. Ribs, lumbar vertebrae, and pelvic bones are fully visible. No fat palpable upon touch. Other bones also show some prominence.
BCS3	Underweight. Ribs are palpable and clearly visible, but no fat is felt upon touch. Upper lumbar vertebrae are

	clearly visible, and pelvic bones are prominently prominent. Significant skin folds are present on the loin and abdomen.
BCS4	Moderate condition with balanced muscle and fat. Ribs are clearly palpable but covered by a thin layer of fat. Viewed from above, the waistline is smoothly defined. Abdominal skin folds are noticeable but not excessive.
BCS5	Ideal body condition with evenly distributed muscle and fat. Ribs are easily palpable without heavy fat coverage. The waistline is clearly visible from above or the side. The abdomen is slightly tucked, with overall smooth lines.
BCS6	Slightly overweight. Ribs are palpable with mild fat coverage. The waist is visible from above but not pronounced. The abdomen is tucked.
BCS7	Extremely thin. From a distance, ribs, lumbar vertebrae, pelvic bones, and entire skeletal structure are clearly visible. Muscle mass is significantly insufficient, with a noticeably thin physique.

Supplemental Table S3. Fecal scoring criteria.

Grade	Criteria
1	Watery diarrhea stool, completely liquid/paste-like (no solid structure).
2	Very soft but not liquid stool, with visible water pockets in different areas compared to Grade 1 stool.
3	Moist stool without cracks, noticeably formed compared to Grade 2 stool; stool sticks together in clumps.
4	Feces are formed with visible cracks, slightly moist on the surface but cohesive. When pressed with a fork, they break apart with resistance and do not flatten as easily as Grade 3 feces.
5	Feces show distinct cracks or form balls, dry both inside and out (Grade 4 feces are dry outside and soft inside). When pressed, the cracked sections separate.

Supplemental Table S4. Tear staining grading criteria and representative photograph.

Grade	Criteria	Representative photograph
1	Discoloration limited to hair near the inner canthus, light in color, dry or moist in texture.	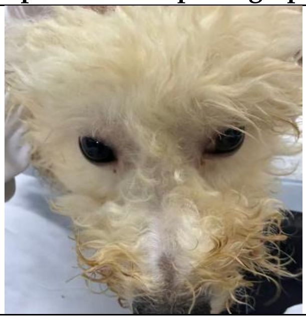

2	Linear discoloration extending from the inner canthus toward the mouth, less than 1 cm in length, pink, light brown, dark brown, or blackish-brown in color, dry or moist in texture.	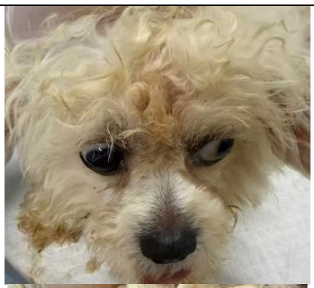
3	Linear discoloration exceeds 1 cm in length, with darker pigmentation and moist texture.	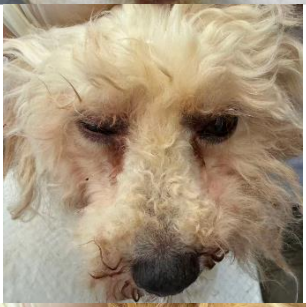
4	Linear discoloration present alongside discoloration of lower eyelid hair from inner to outer canthus, forming a "crescent moon" pattern with darker pigmentation and moist texture.	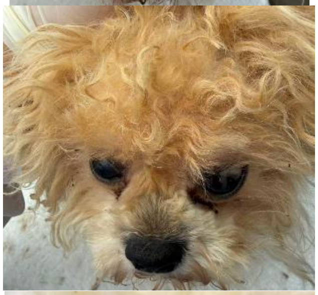
5	Linear discoloration and lower eyelid hair discoloration extend further, forming a "panda eye" pattern. Hair discoloration may even occur within the triangular area bounded by the inner and outer canthi and the corners of the mouth. The color is darker, the texture is moist, and an unpleasant odor may be present.	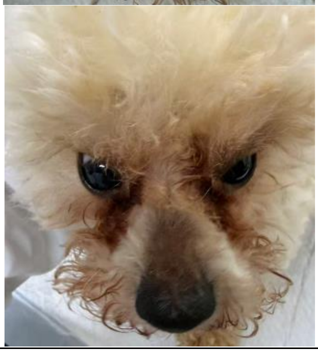

Supplemental Table S5. Effects of dietary supplements on fecal score assessment in experimental dogs.

Days	Control	CM	CS	PC	P value
D0	3.30±0.82	3.80±0.63	3.60±0.52	3.60±0.52	0.380
D7	3.30±0.82	3.60±0.69	3.50±0.53	3.60±0.69	0.744
D14	3.40±0.69	3.40±0.52	3.50±0.53	3.60±0.52	0.838
D21	3.40±0.52	3.40±0.52	3.40±0.52	3.30±0.48	0.961
D28	3.50±0.71	3.60±0.52	3.30±0.48	3.20±0.42	0.349
P value	0.969	0.514	0.742	0.275	

Supplemental Table S6. Effects of dietary supplements on BCS in experimental dogs.

Days	Control	CM	CS	PC	P value
D0	5.30±0.67	5.40±0.84	5.60±0.84	5.40±0.52	0.828
D7	5.35±0.67	5.50±0.71	5.65±0.82	5.45±0.50	0.800
D14	5.40±0.66	5.55±0.68	5.70±0.79	5.50±0.58	0.799
D21	5.45±0.55	5.65±0.67	5.70±0.79	5.55±0.55	0.830
D28	5.50±0.62	5.70±0.71	5.75±0.82	5.55±0.55	0.825

P value	0.960	0.896	0.995	0.964	

Supplemental Table S7. Effects of dietary supplements on body weight in experimental dogs.

Days	Control	CM	CS	PC	P value
D0	3.66±0.99	3.92±1.47	3.86±1.67	3.73±0.73	0.967
D7	3.73±1.07	4.10±1.75	3.93±1.72	3.78±0.83	0.935
D14	3.75±1.08	4.16±1.76	3.98±1.68	3.82±0.83	0.918
D21	3.79±1.06	4.19±1.77	4.03±1.67	3.84±0.83	0.914
D28	3.82±1.14	4.19±1.77	4.03±1.68	3.87±0.80	0.939
P value	0.998	0.996	0.999	0.996	

Supplemental Table S8. Effects of dietary supplements on hematological parameters on Day 28 (the end of the intervention).

Items	Unit	Control	CM	CS	PC	P value
WBC	10^9/L	13.50±3.61	13.90±1.57	12.04±3.13	13.12±2.47	0.495
Lymph #	10^9/L	4.05±1.85	3.96±1.62	4.34±1.81	3.86±1.13	0.922
Monocyte #	10^9/L	0.75±0.21	0.71±0.15	0.77±0.28	0.67±0.25	0.761
Neutrophil #	10^9/L	9.40±3.57	9.43±1.86	8.73±3.48	9.59±3.61	0.935
Lymphocyte %	%	23.30±4.92	24.37±5.03	25.41±3.44	25.64±7.50	0.760
Monocyte %	%	5.56±0.84	5.27±0.88	5.70±1.07	4.78±0.87	0.138
Neutrophil %	%	65.15±11.13	66.86±10.31	61.94±11.18	66.58±11.18	0.737
RBC	10^{12}/L	8.31±1.47	9.01±1.49	7.41±2.36	8.36±1.19	0.227
HGB	g/L	170.00±34.11	184.70±33.40	153.60±46.75	172.50±30.87	0.320
HCT	%	53.41±4.50	54.08±2.86	47.71±9.29	50.31±4.58	0.07
MCV	fL	71.36±3.78	68.07±2.26	70.18±3.05	71.10±2.96	0.085
MCH	pg	20.34±1.02	20.42±0.75	20.81±2.68	20.53±1.07	0.916
MCHC	g/L	285.80±10.95	288.20±7.38	280.70±8.49	285.00±9.38	0.335
RDW-CV	%	15.13±0.96	15.57±0.63	15.89±0.70	15.33±0.68	0.152
PLT	10^9/L	390.70±145.14	458.50±93.21	410.10±211.03	404.50±113.43	0.754
MPV	fL	9.10±0.57	8.85±0.63	9.28±1.08	9.54±0.84	0.287
PDW	-	16.17±0.28	16.10±0.13	16.21±0.28	16.04±0.12	0.312
PCT	%	0.36±0.13	0.41±0.09	0.39±0.19	0.38±0.10	0.867
EOS%	%	3.16±1.35	3.12±1.58	4.36±5.26	2.32±0.61	0.464

[1]**Abbreviation**: WBC, white blood cells; RBC, red blood cells; HGB, hemoglobin; HCT, hematocrit; MCV, mean corpuscular volume; MCH, mean corpuscular hemoglobin; MCHC: mean corpuscular hemoglobin concentration; RDW-CV: red cell distribution width-coefficient of variation; PLT: platelets; MPV: mean platelet volume; PDW: platelet distribution width; PCT: plateletcrit; EOS%: eosinophil percentage.

Supplemental Table S9. Effects of dietary supplements on serum biochemical parameters on Day 28 (the end of the intervention).

Items	Unit	Control	CM	CS	PC	P value
ALB	g/L	34.01±0.74	35.42±2.39	33.67±2.51	34.11±2.03	0.405
TP	g/L	66.61±5.24	70.66±4.71	68.94±5.46	69.72±3.62	0.291
GLOB	g/L	32.64±3.58	35.25±4.62	34.70±4.19	35.61±4.02	0.387
A/G	-	1.05±0.12	1.02±0.17	0.95±0.19	0.97±0.15	0.471
TB	umol/L	0.58±0.69	0.68±0.93	0.43±0.50	0.57±0.57	0.881
GGT	U/L	2.00±0.00	2.50±0.71	2.40±0.70	2.50±1.27	0.465
AST	U/L	46.60±18.57	42.10±9.16	46.90±8.41	39.80±9.78	0.495

ALT	U/L	58.60±20.65	65.40±23.59	55.70±17.99	52.50±13.57	0.496
ALP	U/L	54.10±33.51	48.40±13.13	54.20±40.63	53.50±24.72	0.967
TBA	umol/L	13.61±12.66	11.52±10.35	6.66±7.76	6.84±7.09	0.299
AMY	U/L	813.90±122.90	816.20±235.31	727.80±158.10	730.60±136.86	0.470
LPS	U/L	33.90±10.89	33.80±16.47	37.80±12.22	36.10±12.16	0.886
LDH	U/L	242.90±100.92	266.80±82.66	255.30±133.59	280.80±103.13	0.875
CK	U/L	323.20±111.44	269.80±86.50	376.00±144.31	280.30±78.10	0.132
Creatine	umol/L	61.54±10.19	64.30±8.08	55.62±12.16	55.87±20.14	0.395
UA	umol/L	11.10±3.47	10.10±0.32	10.00±0.00	10.23±0.73	0.504
UREA	mmol/L	6.74±1.35	7.38±2.03	7.38±2.41	7.64±2.01	0.778
U/C	-	112.79±30.33	115.98±33.14	141.35±47.55	162.49±102.46	0.237
GLU	mmol/L	3.73±0.65	3.69±0.98	3.71±0.49	3.49±0.76	0.872
TC	mmol/L	4.30±0.93	4.51±1.38	5.05±1.47	5.22±1.48	0.314
TG	mmol/L	1.17±0.50	0.89±0.31	1.04±0.46	1.09±0.51	0.581
tCO_2	mmol/L	17.16±1.94	17.33±1.04	17.28±0.93	17.76±1.19	0.765
Calcium	mmol/L	2.60±0.12	2.64±0.20	2.62±0.14	2.65±0.24	0.936
PHOS	mmol/L	1.64±0.29	1.65±0.23	1.85±0.42	1.71±0.31	0.463
AST/ALT	-	0.93±0.52	0.75±0.16	0.94±0.36	0.81±0.35	0.600
Ca*PHOS	mmol/L	4.42±1.13	4.36±0.69	4.85±1.16	4.72±1.17	0.692

[1]**Abbreviation**: ALB, albumin; TP, total protein; GLOB, globulin; A/B, albumin/globulin; TB, total bilirubin; GGT, γ-glutamyl transferase; AST, aspartate aminotransferase; ALT, alanine aminotransferase; ALP, alkaline phosphatase; TBA, total bile acids; AMY, amylase; LPS, lipase; LDH, lactate dehydrogenase; CK, creatine kinase; U/C, urea/creatinine ratio; GLU, glucose; TC, total cholesterol; TG, triglycerides; tCO2, total carbon dioxide; PHOS, inorganic phosphorus; Ca*PHOS, calcium × phosphorus product.
